# A Novel Bilateral Data Fusion Approach for EMG-Driven Deep Learning in Post-Stroke Paretic Gesture Recognition

**DOI:** 10.3390/s25123664

**Published:** 2025-06-11

**Authors:** Alexey Anastasiev, Hideki Kadone, Aiki Marushima, Hiroki Watanabe, Alexander Zaboronok, Shinya Watanabe, Akira Matsumura, Kenji Suzuki, Yuji Matsumaru, Hiroyuki Nishiyama, Eiichi Ishikawa

**Affiliations:** 1 Department of Neurosurgery, University of Tsukuba Hospital, University of Tsukuba, 2-1-1 Amakubo, Tsukuba 305-8576, Ibaraki, Japan; anastasiev.alexey.gb@u.tsukuba.ac.jp; 2 Center for Cybernics Research (CCR), Institute of Medicine, University of Tsukuba, 1-1-1 Tennodai, Tsukuba 305-8575, Ibaraki, Japan; 3 Department of Neurosurgery, Institute of Medicine, University of Tsukuba, 1-1-1 Tennodai, Tsukuba 305-8575, Ibaraki, Japan; aiki.marushima@md.tsukuba.ac.jp (A.M.); watanabe.hiroki.gb@u.tsukuba.ac.jp (H.W.); a.zaboronok@md.tsukuba.ac.jp (A.Z.); shinya-watanabey@md.tsukuba.ac.jp (S.W.); yujimatsumaru@md.tsukuba.ac.jp (Y.M.); e-ishikawa@md.tsukuba.ac.jp (E.I.); 4 Ichihara Hospital, 3681 Ozone, Tsukuba 300-3295, Ibaraki, Japan; matsumura.akira.ft@alumni.tsukuba.ac.jp; 5 Artificial Intelligence Laboratory, Center for Cybernics Research, Institute of Systems and Information Engineering, University of Tsukuba, 1-1-1 Tennodai, Tsukuba 305-8573, Ibaraki, Japan; kenji@ieee.org; 6 Center for Cyber Medicine Research, University of Tsukuba, 1-1-1 Amakubo, Tsukuba 305-8575, Ibaraki, Japan; nishiuro@md.tsukuba.ac.jp

**Keywords:** stroke, paresis, upper extremity motor impairment, hand gesture recognition, electromyography (EMG), machine learning, deep learning, CNN-LSTM, neurorehabilitation, healthcare

## Abstract

We introduce a hybrid deep learning model for recognizing hand gestures from electromyography (EMG) signals in subacute stroke patients: the one-dimensional convolutional long short-term memory neural network (CNN-LSTM). The proposed network was trained, tested, and cross-validated on seven hand gesture movements, collected via EMG from 25 patients exhibiting clinical features of paresis. EMG data from these patients were collected twice post-stroke, at least one week apart, and divided into datasets A and B to assess performance over time while balancing subject-specific content and minimizing training bias. Dataset A had a median post-stroke time of 16.0 ± 8.6 days, while dataset B had a median of 19.2 ± 13.7 days. In classification tests based on the number of gesture classes (ranging from two to seven), the hybrid model achieved accuracies ranging from 85.66% to 82.27% in dataset A and from 88.36% to 81.69% in dataset B. To address the limitations of deep learning with small datasets, we developed a novel bilateral data fusion approach that incorporates EMG signals from the non-paretic limb during training. This approach significantly enhanced model performance across both datasets, as evidenced by improvements in sensitivity, specificity, accuracy, and F1-score metrics. The most substantial gains were observed in the three-gesture subset, where classification accuracy increased from 73.01% to 78.42% in dataset A, and from 77.95% to 85.69% in dataset B. In conclusion, although these results may be slightly lower than those of traditional supervised learning algorithms, the combination of bilateral data fusion and the absence of feature engineering offers a novel perspective for neurorehabilitation, where every data segment is critically significant.

## 1. Introduction

Modern healthcare trends are riding the rails of digital transformation, with a growing reliance on information technology, wearable devices, and artificial intelligence (AI) [1,2,3]. A key driver of medical innovation is stroke, which places significant strain on healthcare systems as rehabilitation demands rise to address motor impairments and loss of functional independence. Current data indicate that about one-third of stroke survivors experience residual motor and sensory deficits [3,4,5], a burden that has grown since the COVID-19 pandemic [1,4].

The post-stroke period is particularly critical, as the recovery window is typically confined to the first weeks and months following the event [5]. In addition, clinical manifestations such as post-stroke depression and cognitive impairment further complicate recovery for patients, their families, and healthcare professionals [1,2,4,5].

Particular attention is given to upper extremity function, especially hand dexterity, because it is susceptible to loss after a stroke [2,5,6]. This is important because the hands are essential for most activities of daily living (ADLs) and play a crucial role in determining an individual’s physical function and social participation [4,5,6].

These multifaceted challenges, which often reduce patients’ tolerance for conventional physical therapy, have been partially addressed through broad-spectrum neurorehabilitation strategies that rely on biosignal processing and aim to increase patient engagement [2,7,8]. However, many of these tools come with inherent limitations, such as high cost, lack of portability, and the need for close medical supervision. As a result, the current trend in biosignal processing is shifting towards decentralized rehabilitation, using mobile assistive technologies and AI, particularly deep learning (DL) and other machine learning (ML) algorithms [2,8,9,10,11,12,13,14,15,16,17,18].

In biomedical terms, DL is considered a subset of ML that utilizes multilayer artificial neural networks to analyze and interpret biosignal data from patients [9,10]. Alongside accelerometer sensors, EMG sensors are predominantly used, preferred for their non-invasive nature, relevance to muscle activity, and ease of signal acquisition in both hospital and home settings [8,15,16]. Once acquired and processed, these signals can be used for remote diagnostics and aid recovery through feedback mechanisms, such as controlling exoskeletons or applying functional electrical stimulation (FES) [2,3,7,8].

Deep neural networks (DNNs) have achieved promising results across various domains [9,10], but in the field of EMG-based gesture recognition, only a few architectures are commonly employed: convolutional neural networks (CNNs) and long short-term memory networks (LSTMs) [10,12,16]. These models have shown success, but their performance depends heavily on the amount of labeled data available for training [9,19,20].

CNNs are designed for spatial learning and are widely used in image classification (i.e., two-dimensional grid-organized image data), capturing hierarchical patterns through convolutional and pooling layers. In EMG signal processing, EMG signals are often transformed using wavelet transforms as an input for CNN-based myoelectrical decoding [19,20]. More recently, one-dimensional CNNs have emerged as a distinct approach, better suited for time-series data and requiring less feature engineering. These 1D CNNs are being increasingly studied for EMG decoding across various applications [19]. For instance, Atzori et al. demonstrated the effectiveness of CNNs in classifying hand gestures using EMG signals from the NinaPro database, achieving accuracies comparable to or better than traditional ML classifiers [18]. However, most CNN studies have focused on healthy subjects or, to a lesser extent, on individuals in the chronic stage of stroke or amputees [11,17,18,19].

LSTMs, in contrast, are designed for sequential data processing, leveraging memory cells to capture long-range temporal dependencies [8,21]. Despite their strengths, LSTMs are prone to overfitting and can still face gradient challenges, common pitfalls in learning, especially when computational complexity and training time are not adequately managed [8,10,22]. Additionally, their performance can degrade when processing noisy or irregular signals, which may impair the network’s ability to learn long-term relationships and cause instability during training [21].

Recent research has explored hybrid network architectures combining CNNs and LSTMs to leverage the spatial and temporal features of EMG signals [22,23,24,25,26]. For instance, a CNN-LSTM model achieved a mean recognition accuracy of 90.55% on a dataset of 306 healthy subjects, including EMG signals for five gestures and nine-axis inertial measurement unit (IMU) data [22]. While this approach enables automatic biosignal feature extraction without extensive manual design, it requires segmentation and postprocessing [22,25]. However, its applicability in the subacute stage of stroke remains unclear, as EMG and IMU signals from post-stroke patients are often unreliable due to neuromuscular impairments [2,13,17,27]. Another example involved gesture decoding for a hemiplegic patient, where DNNs assessed upper limb motor states for neurorehabilitation [26].

Given the demand for precise, early, and efficient rehabilitation approaches, and the challenges of unique post-stroke muscle tone patterns for collection and training, as well as the constraints of low-channel signal data for low-latency applications and feature engineering, we leveraged CNNs and LSTMs in a lightweight hybrid network for subacute hand gesture recognition [2,8,22,24]. Based on the literature review, a 1D CNN-LSTM model has not been tested on acute or subacute stroke patients’ muscle signals; previously, such studies have primarily been conducted on healthy individuals [2,8,9].

In this paper, we pioneer the evaluation of a 1D CNN-LSTM model that classifies volitional paretic hand gestures using EMG data from subacute stroke patients. To address the limitations of EMG data collection in early-stage stroke, we introduce a novel bilateral data fusion approach that incorporates signals from both the affected and unaffected upper limbs during training. This architecture captures the spatial and temporal dynamics of diverse paretic EMG signals and remains effective with limited data. Our objective was to test whether DL could leverage atypical EMG patterns to accurately recognize paretic hand gestures. By incorporating bilateral input (i.e., additional training data from non-paretic gestures), our proposed model, optimized through targeted hyperparameter tuning, demonstrates the potential for advancing stroke neurorehabilitation and monitoring.

## 2. Materials and Methods

### 2.1. Data Gathering Outlines

To evaluate the fundamental ADLs essential in the subacute stroke care period [4,5,6,15], seven gestures were selected: rest, hand fist movement (i.e., clenched fist), wrist flexion, wrist extension, fingers opening or spread hand, and two fine movements—index pinch and thumbs up (i.e., upright thumb). The selection of these sets of gestures is justified for several important reasons. First, the resting state was included as a baseline gesture class, as it can provide a comparison for distinguishing the state before and after an intended movement [15,27]. This is particularly important when considering stroke-induced impairments at rest, such as tremors and abnormal muscle synergies, which may result in unintentional movements and complicate the decoding of intended distal hand motions [5,14].

A clenched fist is essential for gripping objects and is one of the fundamental ADLs (e.g., holding a toothbrush). It reflects the functioning of the hand, including both intrinsic muscles and extrinsic muscles, as well as the wrist and forearm, serving as a comprehensive metric of upper limb interconnection [28]. Additionally, a persistent clenched fist is a common early sign of developing upper extremity spasticity and should be addressed for both diagnostic and rehabilitation purposes [29].

Wrist flexion was selected as a key motor movement to assess motor control involving superficial forearm flexors, providing insight into the degree and pattern of post-stroke neuromuscular changes [15]. At the same time, wrist extension, a key component for proper hand positioning during grasping movements, was selected as a discriminative gesture class compared to wrist flexion. This selection is due to the involvement of large superficial forearm muscles, which are accessible via surface EMG electrodes, enhancing the diversity of the suggested hand gestures in stroke patients [13].

The fingers opening (spread hand) gesture was selected to evaluate the function of intrinsic hand muscles, providing insight into a distinct set of hand muscle capabilities compared to gestures that rely heavily on long flexors or involve pinching and gripping [28]. Assessing intrinsic muscle performance is crucial for monitoring and rehabilitating hand paresis, given its importance for fine motor control and tasks required for ADLs, as forearm performance regains more than fine hand movements after a stroke [30].

The index pinch, as a precise, task-oriented gesture, was chosen because it requires a high-precision grip and fine finger movements, and is critical for hand ADLs (e.g., buttoning clothes or using a phone) [29]. Failure to perform this gesture indicates significant limitations in independence and function. Moreover, the index pinch movement is associated with specific metrics which assess hand function and monitor recovery progress post-stroke [5,31].

The upright thumb gesture was chosen because of the thumb’s unique opposable structure, which is crucial for nearly half of the hand ADLs [28,29,31]. Performing this gesture involves specific hand muscles (extensor pollicis longus, extensor pollicis brevis, and abductor pollicis longus) and forearm muscles (e.g., extensor carpi radialis longus). This essential fine motor movement indicates motor impairment after a stroke and serves as a rehabilitation target, reflecting the thumb’s critical role in gripping and pinching and its inclusion in the Fugl-Meyer Assessment for Upper Extremity (FMA-UE) [5].

**Figure 1:**
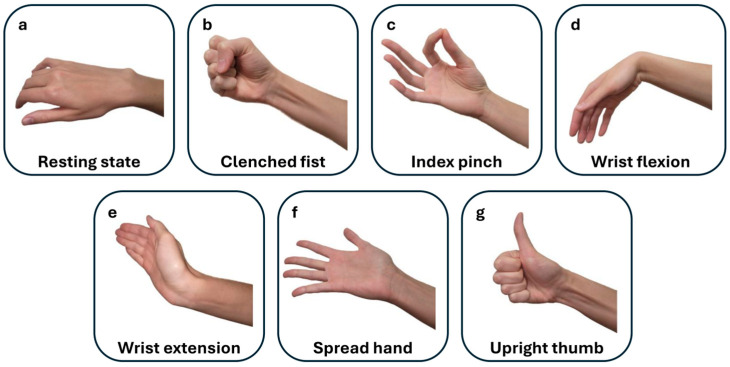
This figure illustrates the selected hand gestures performed during post-stroke movement attempts on both the affected and non-affected sides. The gestures are displayed in the sequential order of the EMG recordings: (**a**) resting state (rest), (**b**) clenched fist, (**c**) index pinch, (**d**) wrist flexion, (**e**) wrist extension, (**f**) spread hand (fingers opening), and (**g**) upright thumb (thumbs up).

Despite the need for extensive research into numerous useful gestures beyond this selection, no additional gestures were studied, as post-stroke patients with a moderate-to-severe status in hospital settings are often medically fragile and face significant physical and cognitive challenges [3,5]. Including unnecessary measurements could be distressing and tiring and could interfere with essential medical care and recovery. The hand gestures studied, illustrated in Figure 1, can also be found in our prior study on supervised hand gesture decoding [14].

EMG data collection was conducted among stroke patients, including those with ischemic and hemorrhagic types of stroke which resulted in upper extremity motor function deficits. The inclusion criteria were as follows: age over 18 years, a registered incident of a cerebrovascular accident within the last eight weeks, and the ability to fully understand the experimental procedure for EMG signal gathering along with a functional assessment [5].

**Figure 2:**
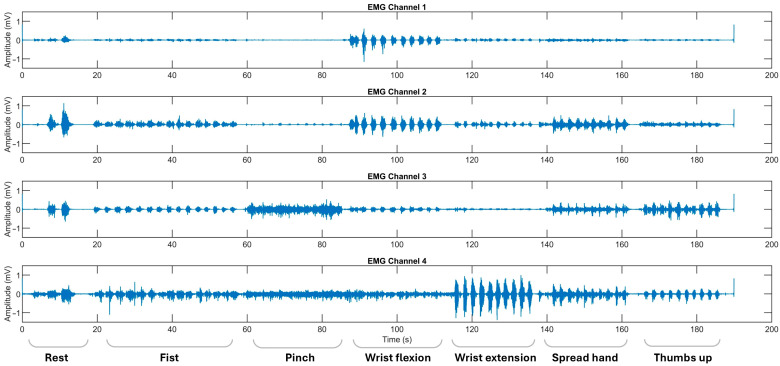
The representative EMG recordings performed by stroke patients using their unaffected upper extremity, with each gesture executed in sequential order. The x-axis represents the recording time in seconds (s), while the y-axis denotes the EMG amplitude in mV. Surface EMG signals were acquired using bipolar electrodes placed over four specific forearm muscle regions: Channel 1 over the flexor carpi radialis, Channel 2 over the flexor carpi ulnaris, Channel 3 over the thenar eminence targeting the abductor pollicis brevis and flexor pollicis brevis, and Channel 4 over the extensor digitorum communis. The initial recording segment captures the pre-calibration and resting state.

**Figure 3:**
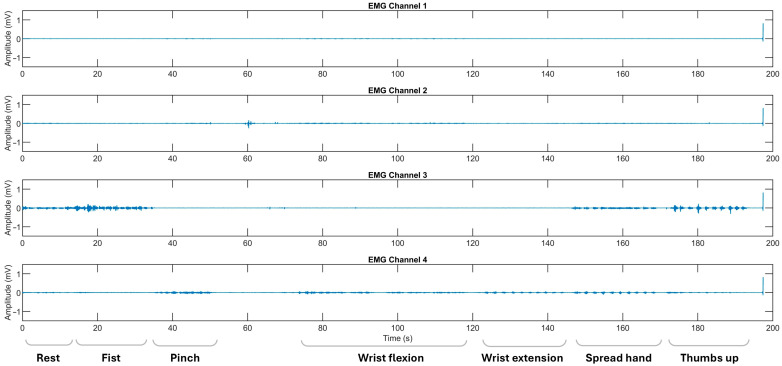
The representative EMG recordings performed by stroke patients using their affected upper extremity, with each gesture executed in sequential order. The x-axis represents the recording time in seconds (s), while the y-axis denotes the EMG amplitude in mV. Surface EMG signals were acquired using bipolar electrodes placed over four specific forearm muscle regions: Channel 1 over the flexor carpi radialis, Channel 2 over the flexor carpi ulnaris, Channel 3 over the thenar eminence targeting the abductor pollicis brevis and flexor pollicis brevis, and Channel 4 over the extensor digitorum communis. The initial recording segment captures the pre-calibration and resting state.

Myoelectrical activities were recorded using an “8CH HUB 19022021” EMG device (PLUX Wireless Biosignals S.A., Lisbon, Portugal) at 1 kHz, with an operative voltage of ±1.5 mV. Ag/AgCl soft electrodes were placed on the flexors and extensors of the forearm and the thenar area of the palm [13,14]. Except for the resting gesture, recorded at the beginning of observational sessions, ADL-related gestures were performed sequentially, with each gesture repeated 10–20 times to ensure a balanced set of 10 gestures per class, providing equivalent per-class EMG signal data for training and testing. Hemiplegic patients repeated hand movements alternately on both extremities (Figure 2 and Figure 3).

Data collection was conducted in hospital settings under two approved clinical protocols of the University of Tsukuba Hospital: R04-041 and R02-204. In study R04-041, recordings from stroke survivors were collected twice with an interval of at least 10 days, whereas in study R02-204, recordings from the same subjects in an observational clinical study were collected only once.

**Figure 4:**
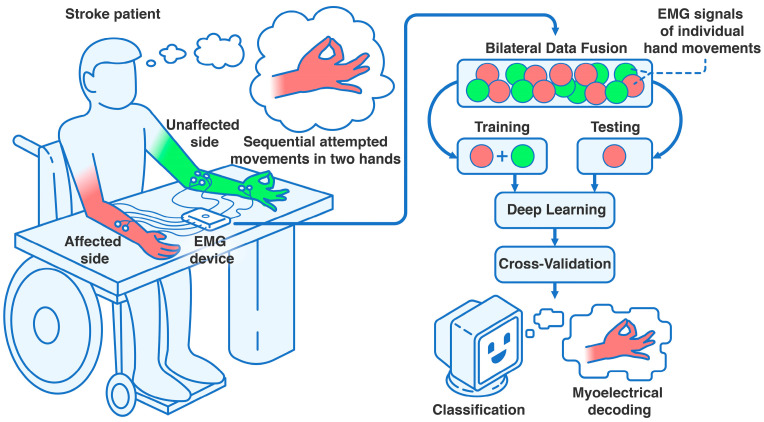
Settings of the clinical trial assessing bilateral data fusion in subacute stroke. Using an 8-channel EMG device (8CH HUB 19022021), four bipolar surface electrode pairs were placed on each forearm and hand. After patients were positioned at the table in the controlled environment, they performed sequential hand movements or movement attempts with their unaffected (i.e., non-paretic) and affected (i.e., paretic) upper extremities, completing at least 10 attempts per gesture class. After recording, the data were processed into an EMG dataset and split into training (data from both extremities) and testing sets (data from the affected extremity only). This was followed by 10-fold cross-validation, iterated 100 times, to correctly classify the intentional paretic gesture for each attempt using DL, leveraging the bilateral data fusion approach.

All recordings, patient selection, and collection of patients’ written informed agreements were guided by the University of Tsukuba Hospital Ethics Committee and Review Board and the Declaration of Helsinki. The Japan Registry of Clinical Trials (jRCT) ID is jRCT1030220380. Details of the study participants can be found in Section 3.1 of the Results. An illustration of the proposed study can be found in Figure 4.

### 2.2. Deep Neural Network Architecture

**Figure 5:**
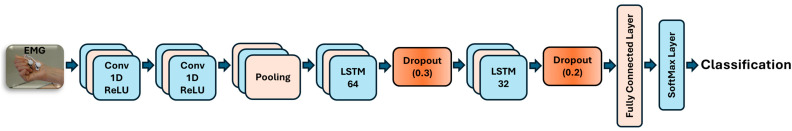
EMG-driven CNN-LSTM neural network architecture for decoding paretic hand gestures.

From each patient, ten repetitions per gesture class were used for the EMG dataset. With a sampling rate of 1 kHz, the signal length of a single gesture attempt was 750 samples. After filtering raw EMG signals with a Butterworth bandpass filter (20–300 Hz), labeling individual gestures (including attempted movements), and normalizing to mean absolute values (with no other feature extraction methods applied), we deployed a 1D CNN-LSTM network for hand gesture classification [22,23,24]. This preprocessing stabilized the learning and validation curves during initial model testing [16]. The network architecture is detailed layer by layer (see Figure 5).

The sequence input layer was configured with an input size of 4 channels, representing features from the 4-channel EMG input. Two 1D convolutional layers were employed, each with a filter size of 6, using 24 filters in the first and 48 in the second layer. Based on empirical tuning, CNN layers were set to use causal padding to maintain the temporal causality of EMG signals, which is crucial given the limited EMG data. Each convolution operation was followed by a rectified linear unit (ReLU) activation and layer normalization to introduce non-linearity and stabilize gradient flow during the network’s learning [11]. Subsequently, a global average pooling layer was added to summarize the temporal dimension of each feature map, reducing computational complexity while preserving key temporal features of the EMG signal in subacute stroke datasets.

Our hybrid network architecture consisted of two LSTM layers for sequence-to-label classification [23]. The first layer had 64 units, and the second had 32 units. Each unidirectional layer was paired with a dropout layer to mitigate overfitting, with rates of 0.3 for the 64-unit layer and 0.2 for the 32-unit layer [24]. The output from the LSTM layers was then passed to a fully connected layer with neurons equal to the number of gesture classes [26]. Finally, a Softmax layer converted scores into probabilities, which were processed by a classification layer for hand gesture recognition [22].

For the training configuration, we employed the Adam optimizer with an initial learning rate of 0.002, using a piecewise schedule that reduced the rate by a factor of 0.1 every 100 epochs [11,12,23]. A gradient threshold of 1 was applied to prevent gradient explosion, and a mini-batch size of 32 was used. The network was trained for up to 300 epochs, with validation checks performed every 150 iterations. To ensure randomness, the training data was shuffled at the beginning of each epoch. All hyperparameters were selected empirically, with reference to related studies [9,10,11,12,16,20,21,22,23,24,25,26].

### 2.3. Model Metrics and Evaluation

**Figure 6:**
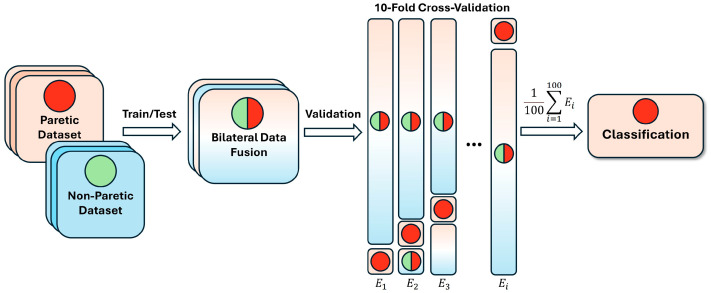
Bilateral data fusion of paretic and non-paretic hand gestures in post-stroke patients. *E*—number of iterations in cross-validation; the green circle represents non-paretic gestures concatenated into subsets; the red circle represents paretic data, randomly selected during cross-validation; the combined circle represents the fused data during training.

To reliably assess the neural network’s performance, we employed k-fold cross-validation using labeled EMG hand gestures from multiple patients [8,13]. The parameters were set to 10 folds and 100 iterations, yielding a mean performance metric for the network (see Figure 6).

For each iteration, data from 23 patients were randomly allocated to the training set and data from 2 patients to the testing set. For the bilateral fusion, all non-paretic data was integrated into the network’s learning process (i.e., non-paretic hand movement data from the same patients was used only for training, not for testing). For the comparison group, paretic data was trained and tested separately without non-paretic EMG data.

For evaluation purposes, we used the sensitivity (SENS), specificity (SP), accuracy (ACC), and F1-score (F1) to assess DNN performance across the paretic and fused EMG gesture datasets [8,26]. SENS, or the true positive rate, measures the ability of DNNs to correctly identify positive instances (e.g., high sensitivity scores indicate that the resting state was accurately classified as rest by the classifier). SP is the proportion of true negatives among all actual negatives, reflecting the model’s ability to avoid misclassifying gestures (e.g., preventing false classification of the resting state as flexion or pinch) [8]. ACC measures the proportion of correctly predicted gestures, reflecting overall performance [2,3,11,15,16,17,18,19,23,26]. F1 quantifies the balance between precision and recall, providing a robust metric for imbalanced datasets common in clinical studies [2,13,21,22]. Therefore, precision scores were not evaluated independently but were considered as components of the F1 metrics.

A statistical comparison was conducted between the performance metrics from the paretic dataset (split into training and testing sets) and the fused dataset, which included non-paretic EMG data from identical gesture classes. Metrics were compared using an unpaired *t*-test, with significance thresholds defined as *p* < 0.05 (significant), *p* < 0.01 (very significant), *p* < 0.001 (highly significant), and *p* < 0.0001 (extremely significant) [14].

To evaluate our DNN’s effectiveness with imbalanced EMG data, we used the Area Under the Receiver Operating Characteristic Curve (AUC-ROC) to assess the performance across gesture classes. The ROC curve plots the true positive rate against the false positive rate across multiple thresholds, demonstrating the model’s discriminative power above chance. The AUC, ranging from 0 to 1 (with 1 indicating perfect classification), offers a single metric of performance per gesture class after cross-validation [8]. Unlike a confusion matrix, the AUC-ROC mitigates misleading interpretations from class imbalance, especially when one gesture dominates, thus providing a balanced evaluation in a multi-class setting. Moreover, the ROC curve enables assessment of the model’s ranking capability by analyzing prediction probability distributions, offering deeper insight into per-gesture classification performance.

Custom scripts for filtering EMG data, learning, testing, cross-validation, and statistical analysis were made and run using MATLAB (R2024b, The MathWorks, Inc., Natick, MA, USA) on a Windows 11 system equipped with an Intel^®^ Core™ i9-7900X CPU, 128 GB RAM, and an NVIDIA^®^ RTX A4000 GPU (16 GB). Additionally, they were executed in a TensorFlow environment (Python 3) on Ubuntu 22.04 using two systems: one with a 13th Gen Intel^®^ Core™ i5-12400, 64 GB RAM, and an NVIDIA^®^ RTX A2000 GPU (6 GB), and another with a 14th Gen Intel^®^ Core™ i5-14500, 64 GB RAM, and an NVIDIA^®^ RTX A2000 GPU (12 GB).

## 3. Results

### 3.1. Post-Stroke Patients

The patient demographics of the combined data from R0-024 (19 patients, one measurement) and R04-041 (6 patients, two measurements) included 25 patients who had experienced either cerebral infarction (CI) or intracranial hemorrhage (ICH), distributed as follows: 12 ICH cases (48.00%) and 13 CI cases (52.00%). Patients S020 to S025 underwent two rounds of assessment, typically spaced two weeks apart, referred to as measurement A (input data for dataset A) and measurement B (input data for dataset B). The overall median time since stroke onset was 16.0 ± 8.6 days for dataset A (R0-024 and R04-041 with the first measurement) and 19.2 ± 13.7 days for dataset B (R02-024 and R04-041 with the second measurement).

The demographic profile of the cohort showed a mean age of 67 ± 12 years, with 18 males (72.00%) and 7 females (28.00%). Due to the complexity of recruitment and frequent discharge to rehabilitation facilities, patients with both dominant and non-dominant affected upper limbs were included: 12 patients had right-sided paresis (48.00%), and 13 had left-sided paresis (52.00%).

On average, EMG signal recording for each patient in the R2-204 trial lasted 30 min, while for those participating in the R04-041 trial, the average recording time was approximately 45 min. The average FMA-UE score across both datasets A and B was 37 ± 20, with a maximum possible score of 66 [4,6]. A detailed summary of patient characteristics is provided in Table 1.

### 3.2. Observed Network Performance

With seven hand gestures available for testing, this study aimed to evaluate the network’s classification capabilities using DL and to examine the effect of bilateral data fusion in EMG signal processing for datasets A and B. In addition to all-in-one gesture prediction (7G-A and 7G-B models), the labeled data were organized into gesture subsets ranging from two to seven classes, enabling assessment across varying levels of classification complexity. To highlight potential real-world applications, gestures were selected to represent distinct muscle activation patterns commonly observed in paretic movements or movement attempts. This subset-splitting strategy is widely used in hand gesture recognition research [9,13,26].

For the two-gesture subsets (2G-A and 2G-B sub-models), the rest state and wrist extension were included. The three-gesture subsets (3G-A and 3G-B sub-models) consisted of rest, wrist extension, and thumbs up. The four-gesture subsets (4G-A and 4G-B sub-models) comprised rest, fist, wrist flexion, and wrist extension. For the five-gesture subsets (5G-A and 5G-B sub-models), rest, index pinch, wrist flexion, wrist extension, and thumbs up were selected. The six-gesture subsets (6G-A and 6G-B sub-models) included all gestures except the fist. Additionally, to address potential limitations associated with the rest state (i.e., its classification may not fully capture distinct movement actions), six-gesture subsets excluding the rest state (6G-NR-A and 6G-NR-B sub-models) were evaluated. The performances of individual gesture labels, based on machine learning metrics, are listed in Supplementary Tables S1 and S2.

The classification performance of the CNN-LSTM model across these gesture sets, along with the results of statistical comparisons between the paretic-only and bilateral fusion datasets, are presented in Table 2 and Table 3.

### 3.3. AUC-ROC Visualization for Paretic and Fused EMG Classification

**Figure 7:**
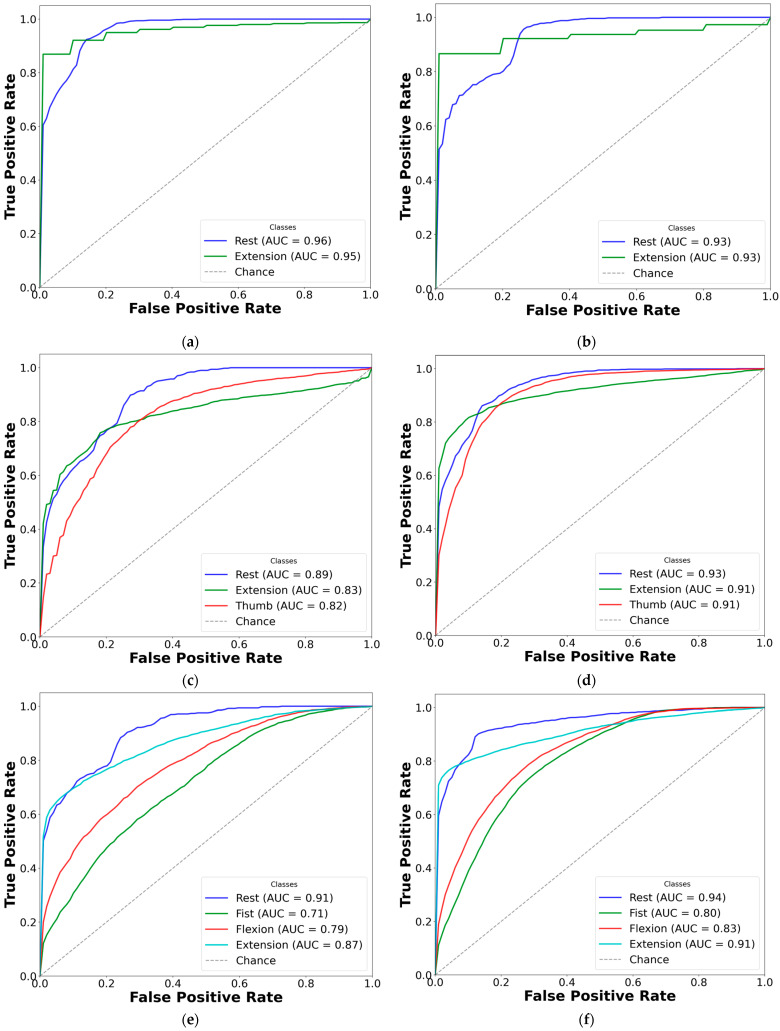

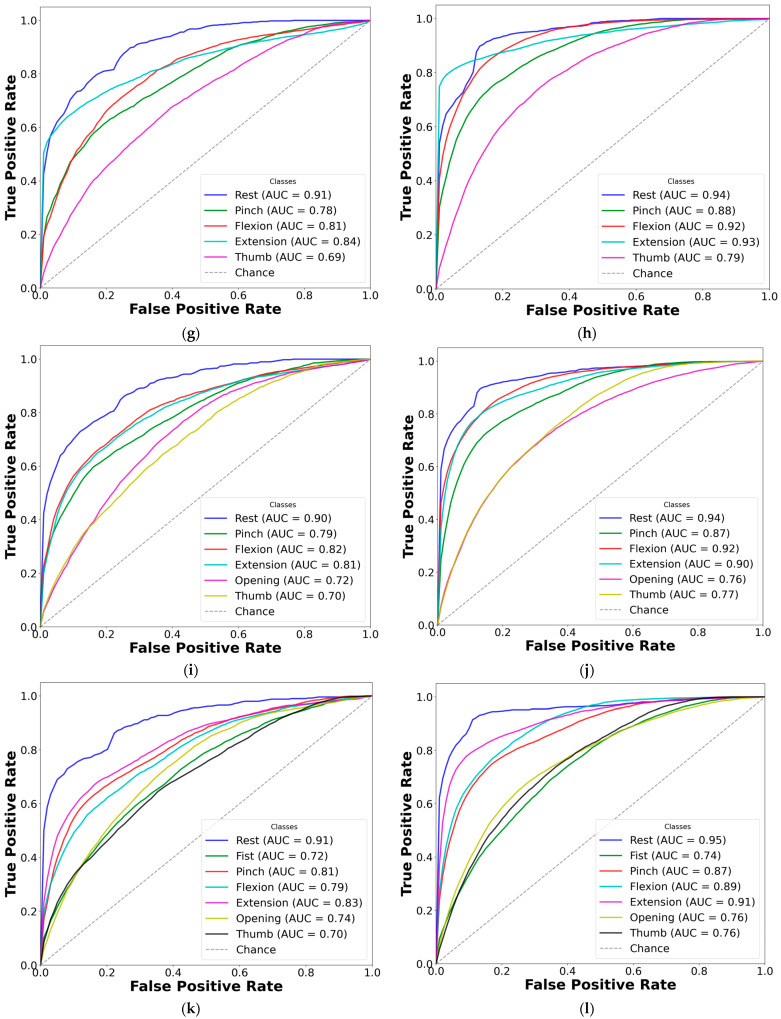

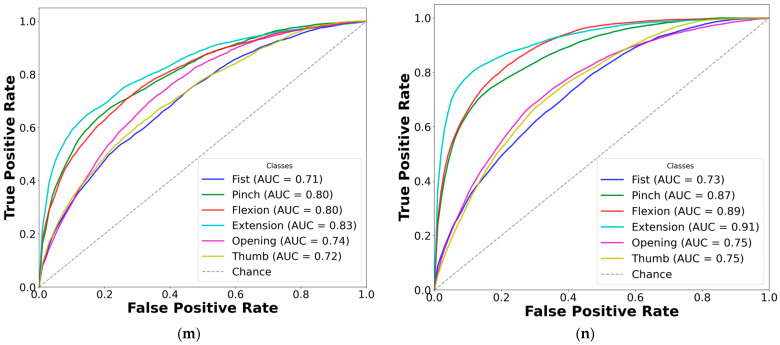
Comparison of AUC-ROC curves from cross-validated EMG hand gesture classification using dataset A. The left plots illustrate cross-validation results using paretic-only data, while the right plots show results from the bilateral data fusion approach. Each ROC curve represents a distinct gesture class within the specified subset: (**a**) two-gesture subset (paretic); (**b**) two-gesture subset (fused); (**c**) three-gesture subset (paretic); (**d**) three-gesture subset (fused); (**e**) four-gesture subset (paretic); (**f**) four-gesture subset (fused); (**g**) five-gesture subset (paretic); (**h**) five-gesture subset (fused); (**i**) six-gesture subset (paretic); (**j**) six-gesture subset (fused); (**k**) all-in-one seven-gesture set (paretic); (**l**) all-in-one seven-gesture set (fused); (**m**) six-gesture subset excluding rest (paretic); (**n**) six-gesture subset excluding rest (fused). AUC—area under the curve, and ROC—receiver operating characteristic curve. The dashed diagonal line represents the baseline of random guessing.

**Figure 8:**
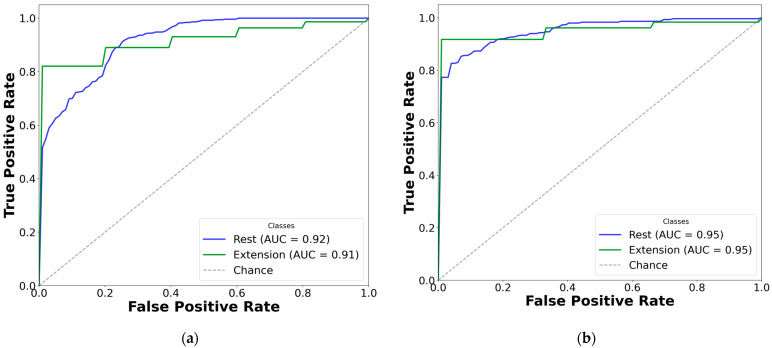

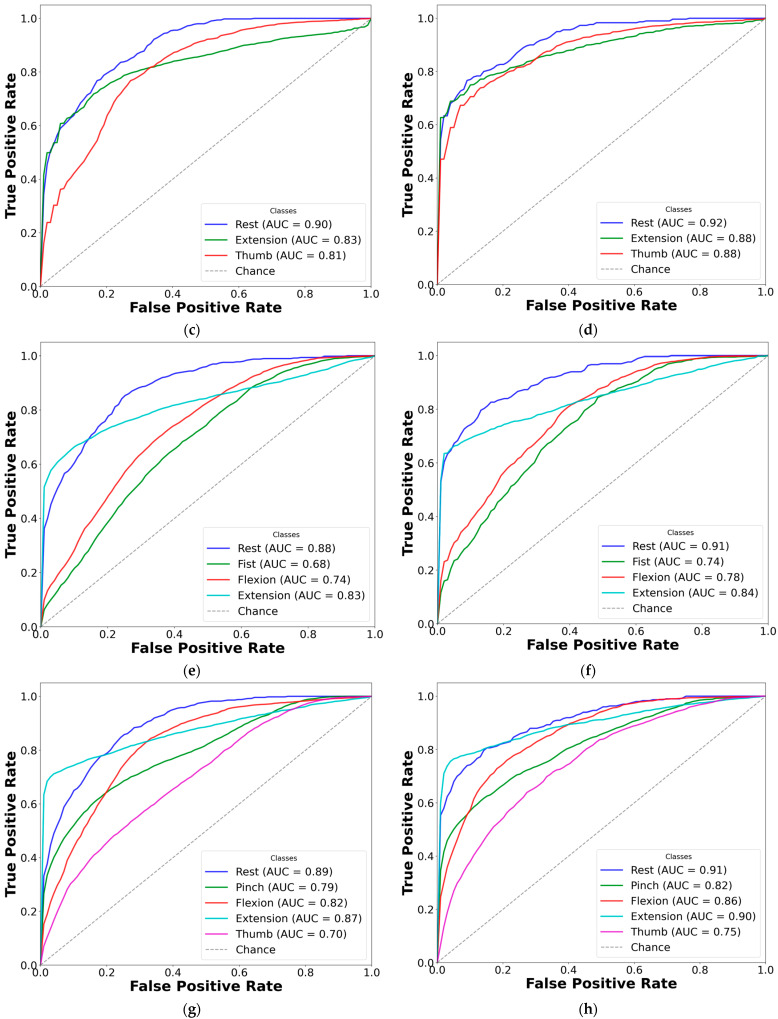

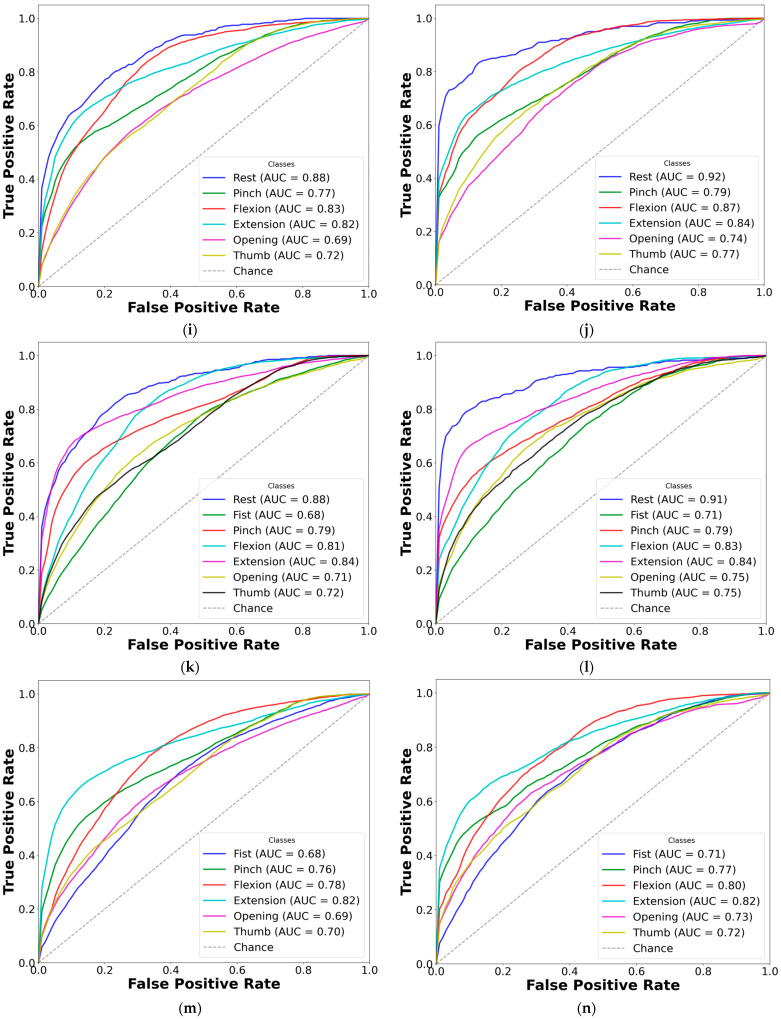
Comparison of AUC-ROC curves from cross-validated EMG hand gesture classification using dataset B. The left plots represent cross-validation results using paretic-only data, while the right plots display results from the bilateral data fusion approach. Each ROC curve corresponds to a distinct gesture class within the specified subset: (**a**) two-gesture subset (paretic); (**b**) two-gesture subset (fused); (**c**) three-gesture subset (paretic); (**d**) three-gesture subset (fused); (**e**) four-gesture subset (paretic); (**f**) four-gesture subset (fused); (**g**) five-gesture subset (paretic); (**h**) five-gesture subset (fused); (**i**) six-gesture subset (paretic); (**j**) six-gesture subset (fused); (**k**) all-in-one seven-gesture set (paretic); (**l**) all-in-one seven-gesture set (fused); (**m**) six-gesture subset excluding rest (paretic); (**n**) six-gesture subset excluding rest (fused). AUC—area under the curve, and ROC—receiver operating characteristic curve. The dashed diagonal line represents the baseline performance of random guessing.

To improve clarity, AUC-ROC plots were used to illustrate the classification performance of each CNN-LSTM network across EMG gesture models. The multi-curve plots are arranged in pairs: the left panels display results for paretic data from datasets A and B, while the right panels represent the corresponding outcomes for the fused datasets. Each ROC curve is shown in a distinct color to represent a specific gesture class, and all plots are derived from cross-validated classification results (see Figure 7 and Figure 8).

Based on the cross-validation results, several important trends were identified in the prediction of paretic gestures, both with and without bilateral data fusion. The results highlight the main observations derived from the comparison of 14 gesture models across datasets A and B (seven paired models per dataset). These findings are supported by the ML metrics (Table 2 and Table 3) and gesture-specific ROC curves with corresponding AUC values (Figure 7 and Figure 8).

First, dataset B demonstrates better performance than dataset A, which includes EMG data from an earlier post-stroke period, with one exception. This trend is reflected in higher SENS, SP, ACC, and F1 metrics in dataset B (see Table 3) and is further supported by the ROC curves and AUC values (Figure 7 and Figure 8). Interestingly, the exceptional case showed that bilateral fusion of the rest and wrist extension gesture pair resulted in significantly lower performance than DNN predictions based solely on paretic data (unpaired *t*-test, *p* = 0.045).

Second, except for one specific gesture pair, bilateral data fusion significantly enhances DNN recognition performance for paretic hand movements. This improvement is statistically validated, with significance levels increasing as the complexity of EMG cluster recognition grows (i.e., when more hand gesture classes are included in the classification). Additionally, the ROC curves for both the paretic-only and fused datasets confirm this trend, showing consistent classification patterns as the gesture class count increases (Figure 7 and Figure 8). Within each gesture subset, the same gestures tend to be dominant or inferior: the rest gesture typically yields the highest AUC, whereas the fist or thumbs up gesture class most often yields the lowest.

Third, an exception to performance gains with increasing gesture classes is observed in specific subsets: rest, wrist extension, and thumbs up in dataset A; and rest, wrist flexion, wrist extension, and fist in dataset B. In these cases, both the paretic and fused datasets exhibit the lowest classification performance among seven pairs of gesture subsets for each dataset. Specifically, the accuracy was 73.01 ± 9.20% (paretic) and 78.42 ± 7.76% (fused) in dataset A, and 77.20 ± 5.82% (paretic) and 78.78 ± 4.31% (fused) in dataset B.

Fourth, both datasets exhibit generally high SP values, ranging from 80% to 89% with a low standard deviation (SD) of 2–3%, except in the described-above two-gesture model (rest and wrist extension). In dataset A, the SP decreased to 66.69 ± 16.53% for paretic data and 64.31 ± 11.9% for fused data; in dataset B, it dropped to 57.09 ± 10.35% for paretic and 62.00 ± 10.79% for fused data.

Fifth, despite accuracy drops for the specific gestures of thumbs up, wrist extension, and rest in dataset A, and rest, wrist flexion, wrist extension, and fist in dataset B, the F1 consistently outperformed other metrics across all gesture classes with bilateral data fusion (compared to paretic-only). These gains were statistically significant, with F1 increases of up to 9% in dataset B and 13% in dataset A.

Finally, excluding the rest gesture class from classification, as tested and validated (see Figure 7m,n, and Figure 8m,n), did not significantly impact CNN-LSTM performance. The results were comparable to the six-gesture model, with both datasets achieving approximately 80% accuracy and stable SENS, SP, and F1 metrics.

## 4. Discussion

As previously described, we split the stroke data into datasets A and B to assess result consistency across different post-stroke time periods independently. This approach allowed us to explore more precisely the conditions under which CNN-LSTM-based classification is most effective. In doing so, we improved the accuracy and reliability of our findings and strengthened the basis for our interpretations and conclusions.

To the best of our knowledge, this study is the first to apply a hybrid CNN-LSTM architecture for decoding volitional hand gestures in subacute stroke patients [2,8,10]. Notably, efficient DL-based classification was achieved with only a limited number of surface EMG sensors on the forearm and hand, sampled at 1 kHz.

To continue, bilateral data fusion for hemiplegic hand gesture recognition, using non-paretic extremity training data, significantly enhanced CNN-LSTM model performance. This improvement is clearly demonstrated in Figure 7 and Figure 8, with the most pronounced performance gain observed in the three-gesture subset, where classification accuracy increased from 73.01% to 78.42% in dataset A (see Table 2) and from 77.95% to 85.69% in dataset B (see Table 3). A visual overview of the method and detailed cross-validation procedures are presented in Figure 4 and Figure 6, respectively. We believe this approach not only improves classification performance but also expands the practical applicability of DNNs in neurorehabilitation.

It is worth mentioning that our EMG classification models, both with and without the proposed bilateral data fusion approach, achieved satisfactory recognition of paretic hand movements across various gesture sub-models in both datasets. The performance levels observed are comparable to those reported in prior CNN-LSTM-based classification studies [22,23,24,26]. As this is the first study to apply DL to subacute myoelectric gesture recognition, direct comparisons with similar post-stroke studies are limited [8,17]. However, parallels can be drawn with related investigations involving chronic stroke survivors, patients with post-traumatic brain injury hemiplegia, amputees, and healthy individuals whose EMG signatures were used in CNN-LSTM models [8,22]. For example, Bao et al. evaluated eight patients in the chronic stage of stroke (1 to 13 years post-onset) using a CNN-LSTM model to control a robotic arm across six hand gestures, achieving an accuracy of 72.81% via intra- and inter-subject transfer learning [10]. In another study, Zea et al. examined a single subject with severe brain trauma using a similar network [26]. Their results highlighted the challenge of generalizing non-paretic gesture recognition to the hemiplegic side, with recognition accuracy as low as 10.67% for the affected limb, compared to 56.00% for the unaffected side. These findings underscore the inherent complexity of paretic gesture recognition across diverse neurological conditions.

Moreover, it is important to emphasize that the EMG signals from the non-paretic side, used in the bilateral fusion approach, should not be considered equivalent to those from healthy individuals. In our study, these signals were collected from the non-paretic upper limbs of subacute stroke patients, who presented with neurological abnormalities such as tremors, ataxia, or uncoordinated motor control [3,13]. On top of that, such patients often face significant mental and physical burdens, including muscle weakness and fatigue, which can limit their ability to perform even basic hand movements with precision [4,5]. However, despite these limitations, our findings, as well as evidence from prior research, suggest that the principle of muscle coactivation remains applicable, since the anatomical structure of the upper limb remains intact [32]. This suggests that EMG signal patterns from the non-paretic side have physiological similarities to those of the paretic side, thus possessing valuable insights for neurorehabilitation strategies [17].

We acknowledge that the CNN-LSTM model developed in this study achieved slightly lower accuracy compared to our previous investigation involving 19 acute and subacute stroke patients, where linear discriminant analysis (LDA) and support vector machine (SVM) classifiers yielded accuracy rates ranging from 89.75% to 90.37% for seven gesture classes [13]. However, several important considerations support the continued value of DNNs and the bilateral data fusion approach. In conventional models, EMG feature engineering for gesture classification in paretic muscles requires substantial manual effort, including the extraction of optimal feature vectors such as wavelet packet coefficients [14]. These feature sets often lack generalizability, particularly in post-stroke populations, due to high inter-individual variability, thus limiting their practical application. In contrast, the CNN-LSTM architecture used in this study (see Figure 5) offers a balanced approach, capturing temporal signal dynamics while maintaining computational efficiency [8,10]. By automatically learning relevant representations from filtered four-channel EMG data, this model provides a more robust and adaptable solution than traditional methods, better suited to the heterogeneity of stroke-related motor impairments [3,14].

For illustration, this explains why models that exclude the rest gesture do not exhibit a significant drop in ROC curve performance (see Figure 7m,n and Figure 8m,n) or in other evaluation metrics (see Table 2 and Table 3). The resting position, being a static gesture, typically yields a low signal-to-noise ratio and minimal muscle activation [27]. Traditional supervised learning approaches, such as SVM, often depend on magnitude-based features (e.g., temporal moments), which are highly susceptible to noise and approximation errors. In contrast, CNN-LSTMs learn more robust feature representations directly from raw or filtered EMG signals, making them less prone to classification loss [13,21,22,26]. This characteristic may account for the consistently high SP scores observed across most models. The lower SP value and higher SD observed only in the two-gesture subset can likely be attributed to the limited amount of training data and restricted number of gesture classes, which constrain the model’s predictive capability.

Regarding the classification performance of the three-gesture subset (3G-A) in dataset A (rest, wrist extension, and thumbs up) and the four-gesture subset (4G-B) in dataset B (rest, wrist flexion, wrist extension, and hand fist), it should be noted that the development of co-contraction in stroke patients often leads to abnormal extensor movements and increased muscle tone [4,32]. These neuromuscular alterations may introduce signal overlaps between gesture classes, thereby affecting classification accuracy [12,24]. Specifically, EMG signals, in terms of voltage and intensity, may become more similar due to the abnormal coupling of flexor and extensor muscle groups. This condition typically results in heightened flexor tone and creates an imbalance in extensor activity, disrupting the normal timing and coordination of agonist–antagonist muscle pairs [32,33]. As a result, signal intensity may decline, and different gestures may become less distinguishable (see Figure 3). These pathophysiological patterns likely contribute to the lower cross-validated AUC-ROC values observed for the fist and thumbs up gestures, particularly in comparison to the rest state (see Figure 7k,l and Figure 8k,l). Consequently, we suggest that such post-stroke motor abnormalities reduce gesture separability, thereby decreasing the predictive reliability of DL models and resulting in lower F1-scores when classifying hemiplegic EMG data.

Overall, the selected gesture subsets (as shown in Table 2 and Table 3) are well-distributed to capture distinct muscle coactivation patterns and bursts across a range of upper limb muscle groups (see Figure 7 and Figure 8), spanning fine motor gestures like thumbs up and index pinch to gross motor movements such as wrist flexion [15]. This systematic approach enables a gradual, detailed evaluation of DNN’s predictive performance, particularly in the context of EMG pattern recognition on the hemiplegic side [10,19].

To sum up, based on the analysis of two datasets and the observed impact of bilateral data fusion in a cohort of 25 stroke patients, we hypothesize that the CNN-LSTM architecture, by combining automated feature extraction through CNNs with the temporal sequence modeling capabilities of LSTMs, holds significant potential for EMG-based gesture recognition. This strength lies in the model’s capacity to generalize across heterogeneous datasets during training, enhancing its adaptability to complex neuromuscular patterns in post-stroke populations [21,22].

We propose that incorporating data augmentation techniques during the training phase, such as introducing EMG data from healthy individuals or participants without neurological deficits, could effectively simulate the benefits of bilateral data fusion (see Figure 4 and Figure 6). This strategy may enhance the model’s ability to predict paretic gestures, even in the acute and subacute stages of stroke recovery, despite the atypical EMG patterns and movement clusters that deviate from normal function which are typically seen during the post-stroke period [13,32,33]. For example, openly accessible datasets such as EMG-EPN-612 or NinaPro may be utilized to merge additional myoelectrical data [2,9,11,17,18,20,22,23,25]. These datasets are important for testing and validating this hypothesis in future research.

Regarding study limitations, the sample size of 25 stroke patients may be insufficient to achieve consistently high F1-scores and accuracy metrics (see Table 2 and Table 3). While this limitation was partially mitigated by incorporating data from the non-paretic side during training, it remains a relevant constraint. In terms of deployment strategies, utilizing the current software and hardware presented additional challenges. The training time for a single CNN-LSTM model, depending on the number of gestures used and data augmentation with unaffected gestures, ranged from 4 to 12 min, while model evaluation required 120 to 180 min per random split. Moreover, employing 10-fold cross-validation iterated 100 times significantly increased the evaluation time, particularly due to the high number of iterations. This method may be necessary for different stroke populations, as the DNN network requires manual tuning to accommodate variability in patient characteristics.

In addition, the network’s parameter configuration was determined empirically and proved highly sensitive. Previous studies have shown that conventional supervised learning classifiers, when combined with feature extraction from well-defined domains, such as data from the non-paretic side, can achieve strong performance [12,13,15]. However, when analyzing EMG data from the hemiplegic side, as demonstrated in our earlier work, predefined feature sets often fail to generalize due to inter-patient variability. In such cases, brute-force or semi-brute-force selection methods are more effective. Therefore, we argue that the application of DNNs is justified, as they provide a more flexible and robust approach in terms of the myoelectrical decoding of hand movements of stroke patients [14].

We believe that the combination of bilateral data fusion and CNN-LSTM-based EMG decoding holds considerable potential for practical applications, particularly in scenarios involving severe paresis or complete paralysis. In bilateral training tasks, the recognition of movement patterns from the non-paretic limb, paired with focused attention on the hemiplegic side, may improve the precision of FES and optimize the operation of exoskeleton-assisted rehabilitation devices [2]. Moreover, the ability to localize temporal intervals of EMG activity during such interventions could enhance rehabilitation outcomes by supporting session planning and promoting patient engagement (which should always be considered a central priority) through real-time visualization of motor gains [7]. This approach also offers the potential for accurate diagnostics and remote monitoring of muscular weakness, facilitating early spasticity detection and enabling the design of advanced, individualized rehabilitation protocols [3,8].

While the classification accuracy achieved in this study is considered acceptable given the degree of motor impairment in the patient population, further improvements are possible. Future research should explore alternative DNNs, such as bidirectional LSTM networks, which may better capture the temporal dependencies in EMG data [25]. Additional efforts will focus on incorporating time-frequency domain feature extraction techniques to enhance model performance, expanding the dataset to include a broader cohort of stroke patients, and validating the model and bilateral data fusion in real-time clinical applications with new participants beyond the current study population [17,20].

## 5. Conclusions

This study presents the first application of a CNN-LSTM-based model for surface EMG signal classification of hand gestures in subacute stroke patients. By introducing a novel bilateral data fusion approach, incorporating non-paretic hand gesture data from the same individuals into the training process, we addressed a major limitation in stroke-specific EMG data availability. Using an all-in-one model with seven gestures, the highest mean accuracy achieved was 83.17%. Furthermore, we identified an optimal subset of gestures yielding a mean accuracy of 88.36%, along with other improved performance scores. The model was evaluated through cross-validation using EMG data from 25 stroke patients (mean time since onset: 16.0 ± 8.6 days for dataset A and 19.2 ± 13.7 days for dataset B; average FMA-UE score: 37 ± 20).

Our results demonstrate that bilateral data fusion significantly enhances the decoding of myoelectrical signals, with the most notable improvement observed in the three-gesture subset (rest, extension, and thumbs up), where accuracy increased by 7.7%. Furthermore, the F1-score, a key metric for evaluating classification performance in imbalanced datasets, showed statistically significant improvements in most scenarios when comparing paretic-only data to fused data. Although the overall accuracy may be lower compared to some canonical supervised learning algorithms, the proposed DL framework provides a compelling advantage by eliminating the need for manual feature engineering, simplifying model development, and more effectively accommodating the complex, lesion-specific nature of stroke EMG data.

## Figures and Tables

**Table 1:** Demographic and clinical characteristics of patients in datasets A and B.

Clinical Trial	Patient No.	Age	Sex	Stroke Type	Onset (Days)	FMA-UE	Paretic Extremity
R02-204 (input data for datasets A and B)	S001	80	M	CI	11	58	R
	S002	32	F	CI	11	50	R
	S003	71	M	ICH	13	64	R
	S004	52	F	CI	8	63	L
	S005	82	M	CI	9	27	L
	S006	81	M	ICH	5	16	R
	S007	77	M	CI	9	60	R
	S008	79	F	ICH	7	28	R
	S009	65	M	ICH	5	55	R
	S010	67	F	ICH	12	37	L
	S011	66	M	CI	13	15	L
	S011	66	M	CI	13	15	L
	S012	64	F	ICH	33	35	L
	S013	63	M	CI	13	59	R
	S014	50	M	CI	19	22	R
	S015	72	F	CI	16	52	R
	S016	57	M	ICH	12	42	L
	S017	57	M	ICH	18	8	L
	S018	64	M	CI	9	60	R
	S019	74	F	CI	12	9	L
R04-041 (measurements from session A were allocated to dataset A, and measurements from session B were allocated to dataset B)	S020-A	75	M	CI	24	10	L
	S020-B				45	10	
	S021-A	74	M	ICH	26	10	L
	S021-B				34	13	
	S022-A	57	M	ICH	25	15	R
	S022-B				34	24	
	S023-A	70	M	ICH	23	55	L
	S023-B				31	56	
	S024-A	80	M	CI	34	47	L
	S024-B				47	54	
	S025-A	51	M	ICH	31	16	L
	S025-B				50	17	

M—male; F—female, CI—cerebral infarction; ICH—intracranial hemorrhage; R—right; L—left); FMA-UE—Fugl-Meyer Assessment of Upper Extremity Scale. Dataset A includes subjects S001 to S019 and the ‘A’ measurements for subjects S020 through S025 (S020A, S021A, etc.). Dataset B includes the same subjects, from S001 to S019, and the ‘B’ measurements for subjects S020 through S025 (S020B, S021B, etc.).

**Table 2:** Mean performance of a CNN-LSTM model classification, averaged across different gesture combinations, on the stroke EMG dataset A.

Model	EMG Type	SENS ± SD (%)	*p*-Value	SP ± SD (%)	*p*-Value	ACC ± SD (%)	*p*-Value	F1 ± SD (%)	*p*-Value
2G-A	Paretic	66.69 ± 16.53	0.2444	66.69 ± 16.53	0.2444	85.66 ± 14.65	0.118	64.05 ± 18.90	0.1008
	Fusion	64.31 ± 11.90		64.31 ± 11.90		82.90 ± 9.64		60.44 ± 11.07	
3G-A	Paretic	49.00 ± 10.08	2.6326 × 10^−12^ ****	76.15 ± 6.57	6.478 × 10^−10^ ****	73.01 ± 9.20	1.1939 × 10^−5^ ****	45.46 ± 13.55	1.5866 × 10^−10^ ****
	Fusion	59.54 ± 9.90		81.82 ± 6.07		78.42 ± 7.76		57.92 ± 12.55	
4G-A	Paretic	47.36 ± 9.99	0.277	84.34 ± 4.21	0.4839	78.37 ± 6.20	0.6892	45.74 ± 11.77	0.0637
	Paretic	48.78 ± 8.40		84.71 ± 3.08		78.68 ± 4.60		48.57 ± 9.63	
5G-A	Paretic	42.91 ± 10.46	2.3245 × 10^−4^ ****	87.12 ± 3.10	0.0483 *	79.93 ± 5.15	0.0287 *	39.28 ± 10.07	1.4523 × 10^−8^ ****
	Fusion	47.57 ± 6.69		87.86 ± 2.05		81.28 ± 3.30		46.71 ± 7.52	
6G-A	Paretic	37.77 ± 8.64	1.2787 × 10^−5^ ****	88.18 ± 2.12	0.0054 **	80.64 ± 3.75	0.0083 **	33.17 ± 8.84	6.543 × 10^−12^ ****
	Fusion	43.24 ± 8.62		89.00 ± 1.99		81.99 ± 3.37		42.68 ± 9.55	
7G-A	Paretic	33.99 ± 8.00	0.0592	89.50 ± 1.59	0.5087	82.27 ± 2.86	0.5768	30.34 ± 7.69	2.3736 × 10^−5^ ****
	Fusion	35.99 ± 6.90		89.64 ± 1.25		82.47 ± 2.26		35.11 ± 7.88	
6G-NR-A	Paretic	40.14 ± 8.85	0.4942	88.04 ± 1.78	0.4910	80.09 ± 2.95	0.5113	36.42 ± 7.99	0.0023 **
	Fusion	40.96 ± 8.13		88.21 ± 1.63		80.36 ± 2.72		40.02 ± 8.51	

The mean average performance metrics are shown as mean ± standard deviation (SD). Asterisks indicate statistical significance using an unpaired *t*-test comparing paretic-only and bilateral fusion datasets: * *p* < 0.05, ** *p* < 0.01, and **** *p* < 0.0001; SENS—sensitivity; SP—specificity; ACC—accuracy; F1—F1-score; 2G-A—sub-model including rest and wrist extension gestures; 3G-A—sub-model including rest, wrist extension, and thumbs up gestures; 4G-A—sub-model including rest, fist, wrist flexion, and wrist extension gestures; 5G-A—sub-model including rest, index pinch, wrist flexion, wrist extension, and thumbs up gestures; 6G-A—sub-model including rest, index pinch, wrist flexion, wrist extension, opening, and thumbs up gestures; 7G-A—sub-model including rest, fist, index pinch, wrist flexion, wrist extension, opening, and thumbs up gestures; 6G-NR-A—sub-model including all gestures except rest (fist, index pinch, wrist flexion, wrist extension, opening, and thumbs up). The prefix “A” indicates that the sub-models are derived from dataset A.

**Table 3:** Mean performance of a CNN-LSTM model classification, averaged across different gesture combinations, on the stroke EMG dataset B.

Model	EMG Type	SENS ± SD (%)	*p*-Value	SP ± SD (%)	*p*-Value	ACC ± SD (%)	*p*-Value	F1 ± SD (%)	*p*-Value
2G-B	Paretic	57.09 ± 10.35	0.0012 **	57.09 ± 10.35	0.0012 **	88.36 ± 12.07	0.0448 *	57.72 ± 14.39	0.156
	Fusion	62.00 ± 10.79		62.00 ± 10.79		84.75 ± 13.17		60.51 ± 13.33	
3G-B	Paretic	52.58 ± 11.38	1.3226 × 10^−10^ ****	79.10 ± 7.79	1.3900 × 10^−12^ ****	77.95 ± 8.30	7.5559 × 10^−12^ ****	52.45 ± 13.79	3.1825 × 10^−6^ ****
	Fusion	63.54 ± 11.48		86.58 ± 6.07		85.69 ± 6.63		65.39 ± 13.17	
4G-B	Paretic	43.81 ± 9.40	2.9325 × 10^−5^ ****	83.43 ± 3.91	0.0156 *	77.2 ± 5.82	0.031 *	42.36 ± 11.05	3.1825 × 10^−6^ ****
	Paretic	48.91 ± 7.34		84.63 ± 2.97		78.78 ± 4.31		49.10 ± 8.69	
5G-B	Paretic	45.08 ± 10.59	0.0119 *	87.44 ± 3.02	0.0241 *	80.84 ± 4.81	0.0287 *	43.23 ± 11.22	2.5175 × 10^−4^ ****
	Fusion	48.51 ± 8.37		88.30 ± 2.26		82.15 ± 3.56		48.70 ± 9.48	
6G-B	Paretic	40.40 ± 11.09	0.0128 *	88.54 ± 2.28	0.0043 **	81.50 ± 3.71	0.0043 **	39.27 ± 11.69	0.0011 **
	Fusion	44.03 ± 9.23		89.42 ± 1.98		82.93 ± 3.25		44.38 ± 10.04	
7G-B	Paretic	32.86 ± 9.22	4.9629 × 10^−5^ ****	89.09 ± 1.66	1.3337 × 10^−4^ ****	81.69 ± 2.83	1.611 × 10^−4^ ****	31.69 ± 9.94	3.8858 × 10^−6^ ****
	Fusion	38.08 ± 8.57		89.97 ± 1.52		83.17 ± 2.60		38.23 ± 2.60	
6G- NR-B	Paretic	38.37 ± 9.74	0.0171 *	87.70 ± 1.94	0.021 *	79.49 ± 3.23	0.0199 *	36.64 ± 9.52	0.0016 **
	Fusion	41.15 ± 6.23		88.24 ± 1.25		80.4 ± 2.08		40.47 ± 6.43	

The mean average performance metrics are shown as mean ± standard deviation (SD). Asterisks indicate statistical significance using an unpaired *t*-test comparing paretic-only and bilateral fusion datasets: * *p* < 0.05, ** *p* < 0.01, and **** *p* < 0.0001; SENS—sensitivity; SP—specificity; ACC—accuracy; F1—F1-score; 2G-B—sub-model including rest and wrist extension gestures; 3G-B—sub-model including rest, wrist extension, and thumbs up gestures; 4G-B—sub-model including rest, fist, wrist flexion, and wrist extension gestures; 5G-B—sub-model including rest, index pinch, wrist flexion, wrist extension, and thumbs up gestures; 6G-B—sub-model including rest, index pinch, wrist flexion, wrist extension, opening, and thumbs up gestures; 7G-B—sub-model including rest, fist, index pinch, wrist flexion, wrist extension, opening, and thumbs up gestures; 6G-NR-B—sub-model including all gestures except rest (fist, index pinch, wrist flexion, wrist extension, opening, and thumbs up). The prefix “B” indicates that the sub-models are derived from dataset B.

## Data Availability

In compliance with Japan’s Act on the Protection of Information (APPI) and instructions received from the Clinical Ethics Review Board of the University Tsukuba Hospital, the obtained data are not available to the public. For further details, please refer to H.K.

## Supplementary Materials

The following supporting information can be downloaded at: https://www.mdpi.com/article/10.3390/s25123664/s1, Table S1. Gesture-specific performance of a CNN-LSTM model on stroke EMG dataset A; Table S2. Gesture-specific performance of a CNN-LSTM model on stroke EMG dataset B.
