# Supplementary material for: A Novel Bilateral Data Fusion Approach for EMG-Driven Deep Learning in Post-Stroke Paretic Gesture Recognition
Source: Sensors (Basel). 2025 Jun 11;25(12):3664. doi: 10.3390/s25123664 (PMC12196683; doi:10.3390/s25123664)
Supplement: Supplementary file 1 [file sensors-25-03664-s001.zip › sensors-3595852-supplementary.pdf]

sensors

MDPI

Article

A Novel Bilateral Data Fusion Approach for EMG-Driven Deep Learning in Post-Stroke Paretic Gesture Recognition

Alexey Anastasiev [1], Hideki Kadone [2*], Aiki Marushima [3], Hiroki Watanabe [3], Alexander Zaboronok [3], Shinya Watanabe [3], Akira Matsumura [4], Kenji Suzuki [5], Yuji Matsumaru [3], Hiroyuki Nishiyama [6] and Eiichi Ishikawa [3]

1 Department of Neurosurgery, University of Tsukuba Hospital, University of Tsukuba, 2-1-1 Amakubo, Tsukuba, 305-8575, Ibaraki, Japan; anastasiev.alexey.gb@u.tsukuba.ac.jp
2 Center for Cybernics Research (CCR), Institute of Medicine, University of Tsukuba, 1-1-1 Tennodai, Tsukuba, 305-8575, Ibaraki, Japan; kadone@ccr.tsukuba.ac.jp
3 Department of Neurosurgery, Institute of Medicine, University of Tsukuba, 1-1-1 Tennodai, Tsukuba, 305-8575, Ibaraki, Japan; aiki.marushima@md.tsukuba.ac.jp (A.Marushima); watanabe.hiroki.gb@u.tsukuba.ac.jp (H.W.); a.zaboronok@md.tsukuba.ac.jp (A.Z.); shinya-watanabey@md.tsukuba.ac.jp (S.W.); yujimatsumaru@md.tsukuba.ac.jp (Y.M.); e-ishikawa@md.tsukuba.ac.jp (E.I.)
4 Ichihara Hospital, 3681 Ozone, Tsukuba, 300-3295, Ibaraki, Japan; matsumura.akira.ft@alumni.tsukuba.ac.jp
5 Center for Cybernics Research, Artificial Intelligence Laboratory, Institute of Systems and Information Engineering, University of Tsukuba, 1-1-1 Tennodai, Tsukuba, 305-8573, Ibaraki, Japan; kenji@ieee.org
6 Center for Cyber Medicine Research, University of Tsukuba, 1-1-1 Amakubo, Tsukuba, 305-8575, Ibaraki, Japan; nishiuro@md.tsukuba.ac.jp
* Correspondence: kadone@ccr.tsukuba.ac.jp

Supplementary materials:

Table S1. Gesture-specific performance of a CNN-LSTM model on stroke EMG dataset A.

Model	Metrics	Gestures used in the hand gesture recognition model						
		Rest	Fist	Pinch	Flexion	Extension	Opening	Thumb
2G-A paretic	Sensitivity	81.20 ± 20.06	-	-	-	88.21 ± 13.70	-	-
	Specificity	88.21 ± 13.70	-	-	-	81.20 ± 20.06	-	-
	F1-score	62.80 ± 22.62	-	-	-	92.15 ± 8.53	-	-
2G-A fused	Sensitivity	84.60 ± 11.32	-	-	-	92.54 ± 7.01	-	-
	Specificity	92.54 ± 7.01	-	-	-	84.60 ± 11.32	-	-
	F1-score	69.38 ± 17.40	-	-	-	95.22 ± 3.76	-	-
3G-A paretic	Sensitivity	21.40 ± 29.16	-	-	-	76.20 ± 15.06	-	75.46 ± 18.17
	Specificity	94.04 ± 10.60	-	-	-	83.54 ± 13.45	-	76.10 ± 13.15
	F1-score	13.89 ± 20.00	-	-	-	78.05 ± 10.74	-	73.79 ± 12.31
3G-A fused	Sensitivity	61.80 ± 18.11	-	-	-	82.87 ± 9.48	-	83.82 ± 10.00
	Specificity	95.68 ± 5.70	-	-	-	89.56 ± 6.99	-	84.57 ± 8.29
	F1-score	55.94 ± 21.15	-	-	-	85.25 ± 5.98	-	83.10 ± 6.32
4G-A paretic	Sensitivity	37.20 ± 31.40	47.76 ± 18.05	-	59.13 ± 19.58	70.65 ± 16.46	-	-
	Specificity	93.86 ± 9.93	79.10 ± 12.81	-	82.25 ± 10.63	86.30 ± 11.68	-	-
	F1-score	27.19 ± 26.52	48.42 ± 13.07	-	59.12 ± 12.68	70.83 ± 11.86	-	-
4G-A fused	Sensitivity	72.10 ± 16.29	61.95 ± 12.26	-	62.53 ± 13.05	77.25 ± 12.45	-	-
	Specificity	95.83 ± 5.19	79.83 ± 8.15	-	83.92 ± 6.79	94.22 ± 4.19	-	-
	F1-score	58.27 ± 22.29	60.17 ± 7.83	-	63.30 ± 8.68	81.21 ± 8.13	-	-
5G-A paretic	Sensitivity	17.20 ± 28.00	-	51.60 ± 20.85	59.31 ± 18.14	66.28 ± 18.86	-	40.10 ± 15.48
	Specificity	95.95 ± 8.79	-	88.08 ± 8.53	84.13 ± 11.79	90.67 ± 10.08	-	80.74 ± 10.31
	F1-score	9.29 ± 15.64	-	53.02 ± 14.29	56.97 ± 13.43	67.87 ± 16.42	-	39.37 ± 10.99
	Sensitivity	58.90 ± 28.42	-	59.53 ± 12.38	77.31 ± 11.28	80.44 ± 11.55	-	50.32 ± 8.54

5G-A fused	Specificity	95.65 ± 5.98	-	91.84 ± 4.18	89.14 ± 7.34	95.84 ± 3.93	-	85.10 ± 5.65
	F1-score	43.96 ± 27.09	-	63.82 ± 8.76	73.80 ± 7.66	83.14 ± 7.50	-	50.62 ± 8.54
6G-A paretic	Sensitivity	16.00 ± 27.27	-	46.59 ± 17.95	62.90 ± 19.87	50.98 ± 21.02	35.18 ± 15.75	32.00 ± 16.21
	Specificity	95.76 ± 9.21	-	91.25 ± 7.24	84.41 ± 12.64	90.42 ± 7.28	85.42 ± 9.38	85.29 ± 8.02
	F1-score	8.12 ± 17.92	-	49.91 ± 13.23	56.25 ± 14.49	52.20 ± 13.80	35.03 ± 11.99	32.03 ± 12.26
6G-A fused	Sensitivity	54.30 ± 28.58	-	59.96 ± 14.66	74.08 ± 12.71	67.67 ± 13.50	39.01 ± 12.41	39.79 ± 11.01
	Specificity	96.44 ± 5.51	-	91.88 ± 3.93	90.22 ± 6.45	93.03 ± 3.46	87.68 ± 6.99	87.12 ± 5.14
	F1-score	42.34 ± 28.10	-	61.29 ± 9.86	69.74 ± 9.08	68.71 ± 8.42	40.73 ± 9.07	40.74 ± 8.91
7G-A paretic	Sensitivity	11.00 ± 24.52	34.66 ± 15.17	43.46 ± 17.65	55.25 ± 16.37	50.27 ± 18.24	36.31 ± 15.80	31.15 ± 14.75
	Specificity	97.30 ± 7.01	87.58 ± 9.14	93.81 ± 4.97	86.13 ± 8.76	92.83 ± 5.51	85.20 ± 8.95	87.49 ± 8.45
	F1-score	4.44 ± 12.80	34.36 ± 11.00	48.10 ± 14.49	49.57 ± 11.88	52.91 ± 13.30	33.96 ± 12.86	31.42 ± 12.99
7G-A fused	Sensitivity	57.30 ± 23.78	36.64 ± 12.67	56.77 ± 12.37	63.49 ± 12.83	67.27 ± 13.24	38.56 ± 11.83	34.22 ± 10.21
	Specificity	97.56 ± 4.24	87.86 ± 5.57	92.94 ± 3.75	90.41 ± 5.30	95.00 ± 2.39	88.36 ± 6.05	88.25 ± 4.43
	F1-score	49.10 ± 23.95	36.23 ± 8.73	58.36 ± 8.52	60.29 ± 8.87	69.42 ± 8.29	38.62 ± 7.85	34.88 ± 8.60
6G-NR-A paretic	Sensitivity	-	32.07 ± 14.22	44.29 ± 18.53	53.52 ± 18.35	53.10 ± 19.87	36.03 ± 17.01	33.92 ± 15.32
	Specificity	-	88.26 ± 8.10	92.40 ± 6.56	86.40 ± 9.65	91.89 ± 5.80	85.85 ± 10.32	85.83 ± 9.94
	F1-score	-	32.91 ± 11.65	47.14 ± 14.17	48.55 ± 13.03	53.95 ± 14.96	33.98 ± 11.39	32.76 ± 11.76
6G-NR-A fused	Sensitivity	-	36.13 ± 11.35	57.12 ± 10.96	64.78 ± 12.67	67.35 ± 11.17	38.35 ± 11.15	37.08 ± 10.99
	Specificity	-	88.24 ± 5.39	93.37 ± 3.38	90.23 ± 5.03	94.76 ± 3.07	87.55 ± 6.19	86.19 ± 6.26
	F1-score	-	36.54 ± 7.77	59.68 ± 8.61	61.10 ± 8.48	69.62 ± 7.61	37.95 ± 8.00	35.62 ± 7.74

* Mean individual gesture-specific performance metrics are shown as mean ± standard deviation (SD) based on 10-fold cross-validation with 100 iterations. 2G-A – sub-model including rest and wrist extension gestures; 3G-A – sub-model including rest, wrist extension, and thumbs up gestures; 4G-A – sub-model including rest, fist, wrist flexion, and wrist extension gestures; 5G-A – sub-model including rest, index pinch, wrist flexion, wrist extension, and thumbs up gestures; 6G-A – sub-model including rest, index pinch, wrist flexion, wrist extension, opening, and thumbs up gestures; 7G-A – sub-model including rest, fist, index pinch, wrist flexion, wrist extension, opening, and thumbs up gestures; 6G-NR-A – sub-model including all gestures except rest (fist, index pinch, wrist flexion, wrist extension, opening, thumbs up). The prefix "A" indicates that the sub-models are derived from dataset A.

Table S2. Gesture-specific performance of a CNN-LSTM model on stroke EMG dataset B.

Model	Metrics	Gestures used in the hand gesture recognition model						
		Rest	Fist	Pinch	Flexion	Extension	Opening	Thumb
2G-B paretic	Sensitivity	85.80 ± 16.15	-	-	-	82.46 ± 16.03	-	-
	Specificity	82.46 ± 16.03	-	-	-	85.80 ± 16.15	-	-
	F1-score	56.07 ± 22.97	-	-	-	88.74 ± 10.64	-	-
2G-B fused	Sensitivity	81.00 ± 23.81	-	-	-	91.13 ± 16.94	-	-
	Specificity	91.13 ± 16.94	-	-	-	81.00 ± 23.81	-	-
	F1-score	71.75 ± 27.57	-	-	-	93.28 ± 13.21	-	-
3G-B paretic	Sensitivity	60.00 ± 29.59	-	-	-	73.50 ± 15.11	-	79.44 ± 13.88
	Specificity	96.71 ± 6.85	-	-	-	82.93 ± 11.61	-	73.91 ± 13.33
	F1-score	30.69 ± 25.75	-	-	-	76.08 ± 11.04	-	75.82 ± 9.35
3G-B fused	Sensitivity	79.44 ± 13.88	-	-	-	77.91 ± 20.10	-	80.68 ± 19.10
	Specificity	94.93 ± 09.45	-	-	-	85.33 ± 16.11	-	82.53 ± 15.84
	F1-score	55.30 ± 29.50	-	-	-	79.58 ± 15.96	-	79.77 ± 15.48
4G-B paretic	Sensitivity	29.60 ± 26.89	43.50 ± 20.38	-	59.20 ± 18.15	66.92 ± 18.39	-	-
	Specificity	96.45 ± 6.95	75.94 ± 12.61	-	73.35 ± 12.37	90.39 ± 8.88	-	-
	F1-score	25.25 ± 23.43	42.62 ± 14.38	-	54.53 ± 11.58	70.72 ± 14.18	-	-
	Sensitivity	49.67 ± 29.01	52.85 ± 19.93	-	60.96 ± 21.93	66.06 ± 21.53	-	-

4G-B fused	Specificity	96.29 ± 8.62	75.77 ± 15.47	-	75.87 ± 15.25	94.12 ± 7.80	-	-
	F1-score	48.44 ± 28.91	50.75 ± 14.40	-	56.79 ± 14.80	72.64 ± 16.94	-	-
5G-B paretic	Sensitivity	18.20 ± 23.11	-	55.01 ± 20.16	69.46 ± 17.77	71.14 ± 17.62	-	37.56 ± 16.56
	Specificity	97.68 ± 6.69	-	88.59 ± 8.02	79.45 ± 13.95	95.53 ± 5.04	-	83.19 ± 9.32
	F1-score	17.73 ± 22.98	-	55.94 ± 13.59	60.56 ± 12.13	76.22 ± 13.36	-	38.38 ± 14.25
5G-B fused	Sensitivity	43.00 ± 24.29	-	49.60 ± 26.82	81.29 ± 15.96	74.30 ± 22.60	-	44.10 ± 19.93
	Specificity	97.26 ± 6.20	-	94.62 ± 5.65	76.74 ± 19.26	97.30 ± 3.75	-	84.66 ± 10.45
	F1-score	44.11 ± 27.08	-	55.00 ± 23.75	67.13 ± 15.04	79.47 ± 17.12	-	44.85 ± 16.58
6G-B paretic	Sensitivity	14.80 ± 24.88	-	45.26 ± 18.55	69.47 ± 17.25	54.65 ± 17.93	33.94 ± 17.83	30.57 ± 14.42
	Specificity	97.13 ± 8.30	-	92.31 ± 5.68	79.74 ± 13.49	91.24 ± 6.57	86.52 ± 8.28	86.86 ± 8.28
	F1-score	9.90 ± 18.12	-	49.31 ± 14.87	56.69 ± 22.97	56.66 ± 13.14	34.57 ± 14.13	32.27 ± 12.72
6G-B fused	Sensitivity	48.33 ± 27.37	-	44.61 ± 24.21	75.06 ± 20.30	61.60 ± 21.44	40.28 ± 20.36	34.23 ± 19.75
	Specificity	98.11 ± 4.55	-	94.98 ± 4.96	80.32 ± 15.25	89.84 ± 9.52	85.50 ± 12.13	90.96 ± 7.93
	F1-score	48.30 ± 28.43	-	50.36 ± 22.53	60.90 ± 16.35	60.21 ± 15.87	39.54 ± 17.40	38.60 ± 19.88
7G-B paretic	Sensitivity	17.60 ± 25.15	25.63 ± 16.23	45.83 ± 18.07	60.33 ± 18.80	59.33 ± 18.71	32.97 ± 15.99	28.74 ± 13.27
	Specificity	98.71 ± 4.37	87.50 ± 8.74	93.07 ± 5.48	81.88 ± 12.13	92.24 ± 5.51	88.56 ± 7.52	88.65 ± 07.48
	F1-score	16.85 ± 23.94	25.04 ± 12.47	49.05 ± 14.04	48.89 ± 12.39	59.07 ± 11.95	33.55 ± 12.83	30.37 ± 11.56
7G-B fused	Sensitivity	43.33 ± 28.62	32.63 ± 22.28	42.69 ± 25.29	61.28 ± 24.13	59.98 ± 24.88	44.79 ± 21.74	25.40 ± 18.25
	Specificity	97.87 ± 4.54	87.06 ± 9.70	95.66 ± 4.42	81.01 ± 15.41	92.68 ± 5.55	85.44 ± 10.36	94.28 ± 5.08
	F1-score	41.09 ± 28.67	30.21 ± 16.60	47.93 ± 23.57	49.05 ± 18.53	58.91 ± 18.46	39.93 ± 16.00	30.73 ± 20.22
6G-NR-B paretic	Sensitivity	-	28.96 ± 16.50	42.46 ± 17.76	54.29 ± 17.97	54.98 ± 19.85	31.62 ± 13.88	29.58 ± 14.27
	Specificity	-	85.46 ± 10.12	92.67± 6.05	81.59 ± 11.45	93.34 ± 04.93	87.90 ± 8.33	87.50 ± 10.70
	F1-score	-	27.37 ± 11.57	45.97 ± 13.61	44.71 ± 10.75	57.08 ± 14.17	32.72 ± 12.27	30.87 ± 13.57
6G-NR-B fused	Sensitivity	-	35.99 ± 20.32	39.73 ± 26.60	55.29 ± 23.73	56.40 ± 22.69	42.28 ± 24.01	26.12 ± 16.45
	Specificity	-	84.95 ± 10.09	96.19 ± 4.49	82.72 ± 14.17	91.42 ± 8.71	84.82 ± 12.24	91.33 ± 9.83
	F1-score	-	32.46 ± 14.39	45.77 ± 25.92	46.72 ± 19.38	55.99 ± 19.33	38.19 ± 18.13	30.56 ± 18.30

* Mean individual gesture-specific performance metrics are shown as mean ± standard deviation (SD) based on 10-fold cross-validation with 100 iterations. 2G-B – sub-model including rest and wrist extension gestures; 3G-B – sub-model including rest, wrist extension, and thumbs up gestures; 4G-B – sub-model including rest, fist, wrist flexion, and wrist extension gestures; 5G-B – sub-model including rest, index pinch, wrist flexion, wrist extension, and thumbs up gestures; 6G-B – sub-model including rest, index pinch, wrist flexion, wrist extension, opening, and thumbs up gestures; 7G-B – sub-model including rest, fist, index pinch, wrist flexion, wrist extension, opening, and thumbs up gestures; 6G-NR-B – sub-model including all gestures except rest (fist, index pinch, wrist flexion, wrist extension, opening, thumbs up). The prefix "B" indicates that the sub-models are derived from dataset B.
